# Electrochemical Determination of Pentachlorophenol in Water on a Multi-Wall Carbon Nanotubes-Epoxy Composite Electrode

**DOI:** 10.3390/s120607033

**Published:** 2012-05-25

**Authors:** Adriana Remes, Aniela Pop, Florica Manea, Anamaria Baciu, Stephen J. Picken, Joop Schoonman

**Affiliations:** 1 Department of Applied Chemistry and Engineering of Inorganic Compounds and Environment, “Politehnica” University of Timisoara, Victoriei Sqr., No. 2, Timisoara 300006, Romania; E-Mails: remes_adriana@yahoo.com (A.R.); aniela.pop@chim.upt.ro (A.P.); anamaria.baciu@chim.upt.ro (A.B.); 2 NanoStructured Materials, Department of Chemical Engineering, Delft University of Technology, Julianalaan 136, 2628BL Delft, The Netherlands; E-Mail: S.J.Picken@tudelft.nl; 3 Materials for Energy Conversion and Storage, Department of Chemical Engineering, Delft University of Technology, Julianalaan 136, 2628BL Delft, The Netherlands; E-Mail: J.Schoonman@tudelft.nl

**Keywords:** multi-wall carbon nanotubes-epoxy composite electrode, electrochemical determination, pentachlorophenol, square-wave voltammetry, cyclic voltammetry

## Abstract

The aim of this study was the preparation, characterization, and application of a multi-wall carbon nanotubes-epoxy composite electrode (MWCNT-EP) with 25%, wt. MWCNTs loading for the voltammetric/amperometric determination of pentachlorophenol (PCP) in aqueous solutions. The structural and morphological aspects of the MWCNT-EP composite electrode were examined by scanning electron microscopy. The electrical properties were characterized by direct-current conductivity measurements in relation with the percolation threshold. The electrochemical behavior of PCP at the MWCNT-EP composite electrode was investigated using cyclic voltammetry in 0.1 M Na2SO4 supporting electrolyte in order to establish the parameters for amperometric/voltammetric determination of PCP. The linear dependence of current vs. PCP concentrations was reached in a wide concentration range from 0.2 to 12 μM PCP using cyclic voltammetry, differential-pulsed voltammetry, square-wave voltammetry, chronoamperometry, and multiple-pulsed amperometry techniques. The best electroanalytical performances of this composite electrode were achieved using a pre-concentration/square-wave voltammetric technique and also multiple-pulsed amperometry techniques envisaging the practical applications. The ease of preparation, high sensitivity, and stability of this composite electrode should open novel avenues and applications for fabricating robust sensors for detection of many important species.

## 1. Introduction

Chlorophenols represent a major group of pollutants of environmental concern. Due to their wide diffusion and toxicologic properties, several chlorinated phenols, such as pentachlorophenol (PCP), 2-chlorophenol, 2,4-dichlorophenol, 2,4,6-trichlorophenol (2,4,6-TCP) have been classified by the United States Environmental Protection Agency as priority pollutants. PCP is the most toxic representative of the chlorophenols and an important organic chemical for environmental studies because of its widespread application in industry, agriculture, and commercial product formation and preservation [1]. It is highly toxic and persistent in water and soil, and PCP concentrations in various surface waters from different countries ranging from trace levels to 10,500 μgL^−1^ have been reported by the World Health Organization [2]. Furthermore, it can accumulate in living organisms and result in negative effects, including carcinogenicity and acute toxicity.

Based on the above-presented considerations, it is highly desirable to develop sensitive and convenient technology for the determination of PCP. Various methods have been developed to detect PCP in contaminated samples, most of them based on gas chromatography (GC) [3,4], gas chromatography–mass spectrometry (GC–MS) [5], high performance liquid chromatography (HPLC) [6], thin-layer chromatography [7], and spectrophotometry [8]. Among these, the electrochemical methods have attracted substantial attention because of proven simplicity, sensitivity, selectivity, fast response, and low-cost production. Several studies have reported the electrochemical detection of PCP based on a carbon-paste electrode and vitreous carbon surfaces [9,10].

In our previous work, the results of PCP determination using a carbon nanofiber composite electrode were reported [11]. In order to improve the electroanalytical performance for the determination of PCP, the carbon nanofiber was replaced with carbon nanotubes (CNT), taking into account that carbon nanotubes offer exciting possibilities for developing a sensitive electrochemical sensor because of their excellent properties, such as high electrical conductivity, good chemical stability, and extreme mechanical strength [12–14].

In this work, the electrochemical methodology for the determination of PCP in aqueous solutions using a multi-wall carbon nanotubes-epoxy (MWCNT-EP) composite electrode and various electrochemical techniques, *i.e.*, cyclic voltammetry (CV), differential-pulsed voltammetry (DPV), square-wave voltammetry (SWV), chronoamperometry (CA), and multiple-pulsed amperometry (MPA) is described. Using all these techniques, the electroanalytical parameters for the determination of PCP in the concentration range of 0.2–12 μM are determined. This work offers a new approach to the synthesis of a stable MWCNT-EP composite electrode with excellent electrochemical properties and very attractive for electrochemical studies and electroanalytical applications.

## 2. Experimental Section

### 2.1. Materials

Multi-wall carbon nanotubes (MWCNTs, purity >90%, diameter around 9.5 nm, length around 1.5 μm, and surface area around 250–300 m^2^/g, according to the specifications of the provider) synthesized by catalytic carbon vapour deposition (CCVD) were purchased from NanocylTM, Belgium. Araldite®LY5052 and its corresponding hardener Aradur®5052 which were obtained from Huntsman Advanced Materials, Switzerland, were used as polymeric matrix. Tetrahydrofuran (THF) used as dispersing agent was purchased from Sigma-Aldrich BV. Pentachlorophenol (PCP), sodium hydroxide (NaOH), and sodium sulfate (Na_2_SO_4_) were analytical reagent grade from Merck. The MWCNTs and all the reagents were used as received.

### 2.2. Composite Preparation

The MWCNT-EP composite material was obtained by an effective two-roll mill (TRM) method of a two-component MWCNTs mixed with epoxy resin (Araldite®LY5052/ Aradur®5052). The ratio of the components was selected to obtain 25%, wt. content of MWCNTs and 75%, wt. content of epoxy resin. In the first step, MWCNTs without any further treatment were dispersed into THF by ultrasonication using a Cole-Parmer® 750-Watt Ultrasonic Processor for a specific period of time (10 min) to spread out the nanotubes prior to mixing with the epoxy resin. The second step in achieving a high level of dispersion was to mix the suspension and the liquid epoxy resin (without hardener). The mixture was degassed in a vacuum oven at 60 °C for about 12 hours to remove the solvent. In the processing step, the batch of MWCNT and epoxy resin was two-roll milled for several times on a laboratory scale two-row mill (Collin) at constant temperature of 70 °C and also at different times and shear intensities. Then the hardener was added and mixing was continued for an additional 10 min to ensure a uniform homogeneity. The composite paste was poured into cylindrical PVC tubes, electrical contact was assured using a copper wire and was cured in an oven at 80 °C for 24 h, after which it was left to cool down at room temperature for 24 h.

### 2.3. Characterization of the Composite

#### 2.3.1. Dispersion Quality

The dispersion degree of the MWCNTs in the tetrahydrofuran (THF) solvent was assessed by Dynamic Light Scattering (DLS) also known as Photon Correlation Spectroscopy, performed on a Malvern Instruments Limited Zetasizer Nano-ZS, using the 173° angle Non-Invasive Back-Scatter mode and the M3-Phase Analysis Light Scattering mode. The instrument used a 4.0 mW 633 nm He-Ne laser. The multiple peak high-resolution fitting procedure was used to determine the particle size distribution from the auto-correlation function. About 0.5 mg of MWCNTs was dispersed by ultrasonication using a Cole-Parmer^®^ 750-Watt Ultrasonic Processor for about 10 minutes in THF.

#### 2.3.2. Scanning Electron Microscopy

Morphological characterization of the MWCNT-EP composite electrode was carried out using scanning electron microscopy (SEM XL20, Philips) with an acceleration voltage of 15 kV.

#### 2.3.3. Electrical Conductivity

The electrical conductivity of the MWCNT-EP composite electrode was determined by four-point probe contact (DC) conductivity measurements. All measurements were performed using a digital multimeter DMM2000 and a current source 6221 DC, both provided by Keithley. Silver paste was used as electrical contacts.

#### 2.3.4. Electrochemical Measurements

Electrochemical measurements were performed in unstirred solutions using a computer controlled Autolab potentiostat/galvanostat PGSTAT 302 (EcoChemie, The Netherlands), with a standard three-electrode configuration. The three-electrode system consisted of a MWCNT-EP working electrode with 0.196 cm^2^ geometrical area, a platinum wire as counter electrode and a saturated calomel reference electrode (SCE). Before each voltammogram, the MWCNT-EP composite electrode was carefully polished with abrasive paper and subsequently on a felt-polishing pad by using 0.3 μm alumina powder (Metrohm, Switzerland). The electrode was then sonicated for 5 min in pure water. All experiments were carried out using a standard cell with 50 mL of solution at room temperature (25 °C).

An aqueous 10 mg/L PCP stock solution was prepared daily by dilution the solid PCP in double distilled water and 0.1 M NaOH. The supporting electrolyte for the characterization and application of electrode material in the detection process was a 0.1 M Na_2_SO_4_ solution, which was freshly prepared from Na_2_SO_4_ of analytical purity (Merck) with double distilled water.

## 3. Results and Discussion

### 3.1. Dispersion Assessment of MWCNTs in THF

**Figure 1.**
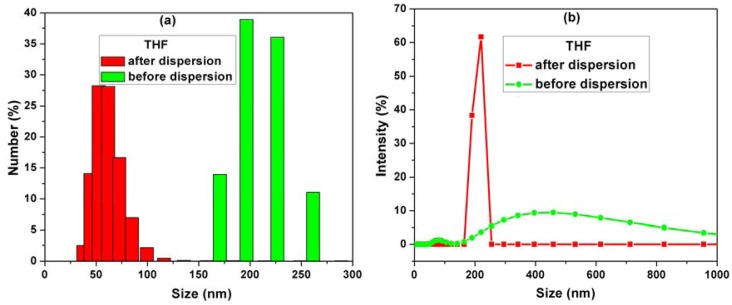
(**a**) Size distribution of MWCNTs before and after dispersion in THF. (**b**) Intensity of MWCNTs before and after dispersion in THF.

Figure 1(a,b) shows the histograms of MWCNT sizes measured by a particle size analyzer before and after dispersion in THF solvent.

By comparison of the MWCNTs sizes before and after dispersion in THF, the information about the dispersion degree in THF is obtained. Before dispersion the mean particle sizes ranged between 150–300 nm. After dispersion, the mean particle sizes revealed more than 50% decrease in agglomeration of CNTs as a result of the ultrasonication process (Figure 1(a)).

Also from the intensity distribution graph (Figure 1(b)), it can be seen that the intensity line is shifting to a narrow size distribution in comparison with the non-dispersed CNTs, for which the intensities are spread over the range 200–800 nm. These results prove that the average particle sizes were decreased by dispersion because the MWCNTs agglomeration is reduced.

### 3.2. Morphological Characterization

**Figure 2.**
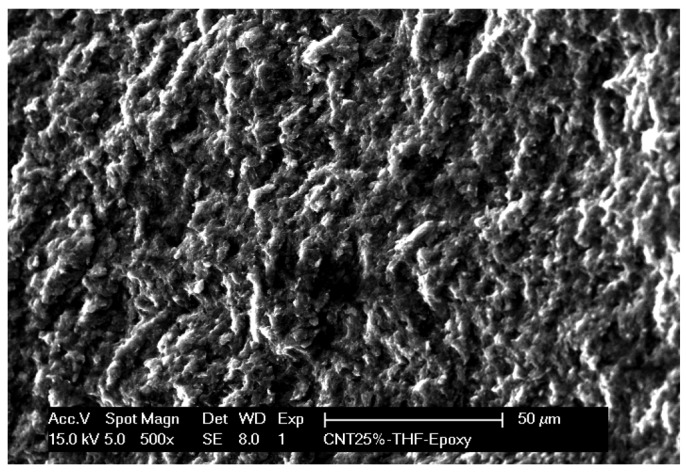
SEM image of MWCNT-EP composite electrode.

The dispersion and bonding of the nanotubes to the epoxy resin matrix is the most important issue in producing the CNT-epoxy composite materials [15]. SEM microscopy was used to gain insight into the surface characteristics of the MWCNT-EP composite. Figure 2 shows the SEM image of the MWCNT-EP composite electrode and it can be seen that MWCNTs are homogeneously dispersed and distributed within the polymer matrix.

### 3.3. Electrical Characterization

**Figure 3.**
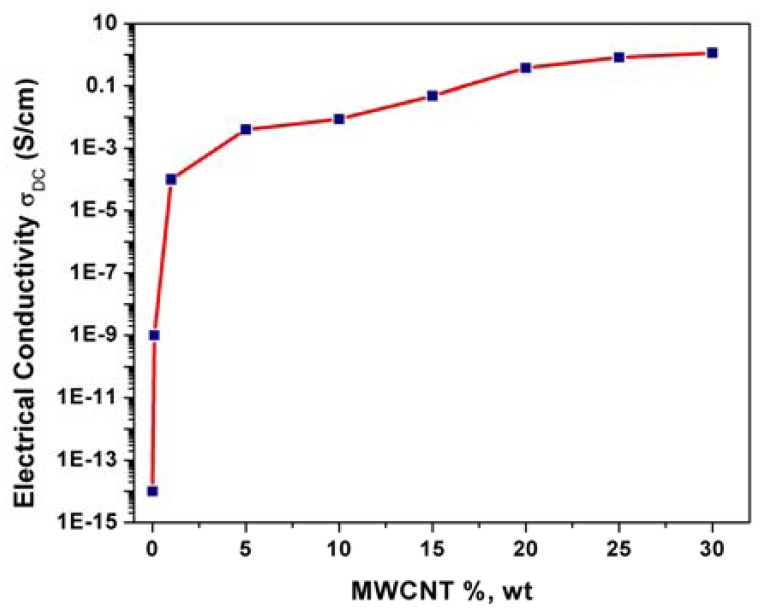
Four-point probe electrical conductivity as a function of MWCNTs content within epoxy matrix.

The electrical conductivity of a composite is strongly dependent on the filler loading and Figure 3 shows the evolution of the electrical conductivity with MWCNTs loading within the epoxy matrix. At low filler concentrations, the conductivity remains very close to the conductivity of the pure, electrically insulating polymer matrix since the fillers are dispersed individually or are present as small clusters in the matrix. The electrical conductivity increased with the MWCNTs loading increasing until a certain value, which represents the percolation threshold concentration. For this MWCNTs loading range, the independent CNT fillers tend to link together to form conductive networks, leading to a significant increase in the electrical conductivity of the composite (from 10^−9^ S/cm to 10^−4^ S/cm). Above this MWCNTs loading value, no significant increasing of the electrical conductivity occurred and a plateau is reached.

A loading of 25%, wt. MWCNTs was selected as optimal composition for further composite preparations based on the correlation between morphology and electrical conductivity that affects significantly the electrochemical behavior.

### 3.4. Electrochemical Characterization

Cyclic voltammetry (CV) is one of the most versatile electrochemical techniques used in the study of electroactive behaviour and the characterization of sensors. In order to determine the electroactive surface area of the MWCNT-EP composite electrode the electrochemical behaviour of potassium ferrocyanide K_3_[Fe(CN)_6_] in 1 M KNO_3_ supporting electrolyte was studied using cyclic voltammetry recorded at different scan rates. According to the Randles–Sevcik Equation (1):

(1) $$I_{p}=2.69\times 10^{5}{\text{AD}}^{1/2}n^{3/2}v^{1/2}C$$

where A represents the area of the electrode (cm^2^), n the number of electrons participating in the reaction, and is equal to 1, D the diffusion coefficient of the molecule in solution, C the concentration of the probe molecule in the solution, and is 4 mM, and v is the scan rate (Vs^−1^). The apparent diffusion coefficient of K_3_[Fe(CN)_6_] was determined to be 5.33 × 10^−6^ cm^2^s^−1^. By comparison with the theoretical diffusion coefficient value of 6.7 × 10^−6^ cm^2^s^−1^ based on the literature data [16], the value of the active electrode area was found to be 0.173 cm^2^ *vs.* the value of the electrode geometric area of 0.196 cm^2^.

**Figure 4.**
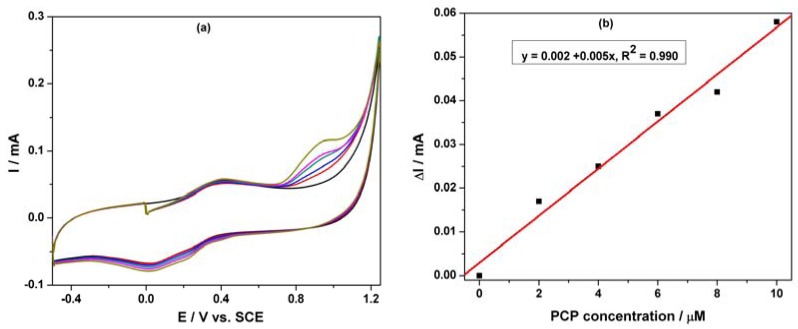
(**a**) Cyclic voltammograms of the MWCNT-EP composite electrode recorded at a potential scan rate of 0.05 Vs^−1^ and a potential range between ^−^0.5 and +1.25 V/SCE in 0.1 M Na_2_SO_4_ supporting electrolyte (curve 1) and in the presence of different PCP concentrations: (2) 2 μM; (3) 4 μM; (4) 6 μM; (5) 8 μM; (6) 10 μM. (**b**) The calibration plot of the anodic currents recorded at E = +0.97 V/SCE *vs.* PCP concentration.

Based on our previous research regarding the electrochemical determination of pentachlorophenol (PCP) on a carbon nanofiber-expanded graphite-epoxy composite electrode [11], the electroanalytical peculiarities of this MWCNT-EP composite electrode towards PCP determination were studied. Cyclic voltammograms (CVs) recorded on the MWCNT-EP composite electrode in 0.1 M Na_2_SO_4_ supporting electrolyte and various concentrations of pentachlorophenol (PCP) are shown in Figure 4(a).

In the absence of PCP, a first anodic current peak is noticed on the CVs around +0.40 V *vs.* SCE, with the corresponding cathodic one is recorded at 0 V *vs.* SCE, due to the oxido-reduction process of the MWCNTs. Some interactions between functional groups of the MWCNT-EP surface and PCP are possible, which precede the oxidation process at more positive potential. The oxidation process of PCP on the MWCNT-EP composite electrode occurred at the potential value of about +0.97 V *vs.* SCE, and only one peak appeared. The current corresponding to the peak oxidation of PCP, increased progressively with its concentration. On the following reverse scan from +1.25 V to −0.5 V *vs.* SCE, no corresponding reduction peak is observed within the potential range between +1.25 and +0.40 V *vs.* SCE, revealing that the anodic PCP oxidation on the MWCNT-EP composite electrode is totally irreversible. The current densities corresponding to the anodic oxidation peaks recorded at + 0.97 V *vs.* SCE increased linearly with PCP concentrations with a correlation coefficient of 0.990 (Figure 4(b)). In general, a proportional increase of anodic current with concentration gives information about the possibility of a controlled oxidation process by mass transfer [17], the desired behaviour for the potential amperometric/voltammetric detection application.

**Figure 5.**
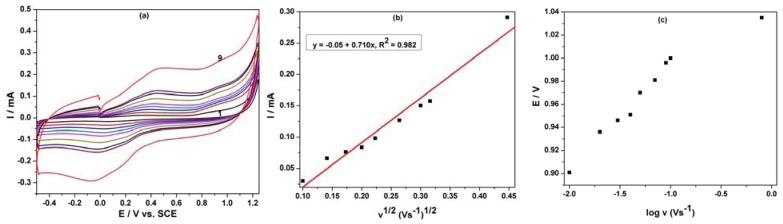
(**a**) Cyclic voltammograms of 8 μM PCP at the MWCNT-EP composite electrode in 0.1 M Na_2_SO_4_ supporting electrolyte (1) with different scan rates: 0.01, 0.02, 0.03, 0.04, 0.05, 0.07, 0.09, 0.1, 0.2 Vs^−1^; potential range: –0.5 and +1.25 V/ SCE. (**b**) The anodic peak current *vs.* square root of scan rate. (**c**) The peak potential Ep *vs.* log (v).

The oxidation process of phenol derivatives on carbon-based electrodes is a very complex process, involving both the adsorption of the reactant/intermediate or phenol oxidation products and the formation of passive, nonconductive layers of oligomer products of the oxidation process on their surface by electropolymerization [11,18]. The cyclic voltammetry of the MWCNT-EP composite electrode at various scan rates (0.01–0.2 Vs^−1^) in the presence of 8 μM PCP was studied (Figure 5(a)) to elucidate the mechanistic aspects of the overall oxidation process of PCP on the electrode surface. For the whole range of the scan rates studied, the peak shape that is sensitive to the scan rate shows irreversible characteristics. The anodic current recorded at about +0.97 V *vs.* SCE increased linearly with the square root of the scan rate (Figure 5(b)) suggesting a mass-transfer controlled process. Moreover, the starting potential of the wave shifted towards positive potential when increasing v indicating that the electrooxidation process of PCP is irreversible (Figure 5(c)).

### 3.5. Pulsed-Voltammetric Measurements

Differential-pulsed and square-wave voltammetric techniques are widely studied to improve the electroanalytical parameters for the voltammetric detection, e.g., the lowest limit of detection and the sensitivities. Also, they can provide information about mechanistic aspects regarding the PCP electrooxidation process.

**Figure 6.**
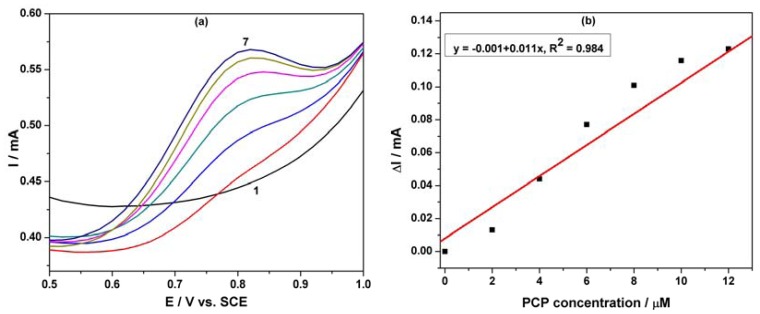
(**a**) DPVs of the MWCNT-EP composite electrode at (modulation amplitude 0.2 V, step potential 0.02 V) a potential scan rate of 0.05 Vs^−1^ in the potential range between +0.5 V and +1.0 V *vs.* SCE in 0.1 M Na_2_SO_4_ supporting electrolyte (curve 1) and in the presence of different PCP concentration: (2) 2 μM; (3) 4 μM; (4) 6 μM; (5) 8 μM; (6) 10 μM; (7) 12 μM. (**b**) Calibration plot of the anodic currents recorded at E = +0.80 V/SCE *vs.* PCP concentration.

Differential-pulsed voltammetry (DPV) has been employed as a technique for the evaluation of the performance of the MWCNT-EP composite electrode for the determination of PCP. The effects of DPV scan parameters on the response of the MWCNT-EP composite electrode have been studied. Modulation amplitude (MA) and step potential (SP) parameters have been considered to optimize the determination method and the best results were achieved for MA of 0.2 V and SP of 0.02 V. Figure 6(a) shows the DPVs recorded for the PCP concentration ranged between 2 and 12 μM in the potential range of +0.5 to +1.0 V *vs.* SCE, and the oxidation potential is shifted to the negative direction in comparison with the CV results (+0.80 V *vs.* +0.97 V *vs.* SCE). The useful net current signals corresponding to the oxidation peak recorded at +0.80 V/SCE are linearly dependent on the PCP concentration (Figure 6(b)). The lower detection potential value and a better sensitivity were achieved by using DPV in comparison with CV (see Table 1).

**Figure 7.**
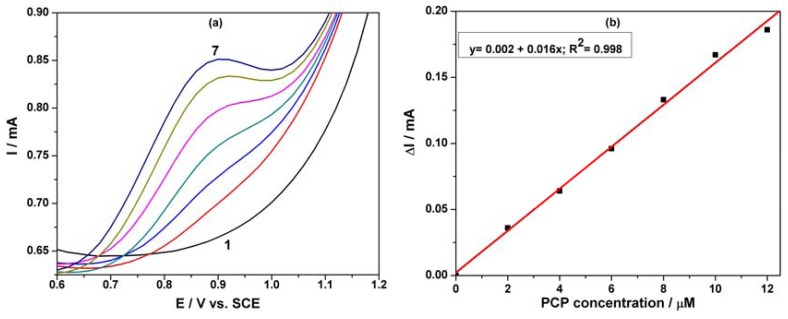
(**a**) SWVs of the MWCNT-EP composite electrode (modulation amplitude of 0.1 V, step potential of 0.01 V and frequency 10 Hz) in the potential range between +0.6 V and +1.2 V *vs.* SCE in 0.1 M Na_2_SO_4_ supporting electrolyte (curve 1) and in the presence of different PCP concentrations: (2) 2 μM; (3) 4 μM; (4) 6 μM; (5) 8 μM; (6) 10 μM; (7) 12 μM. (**b**) Calibration plot of the anodic currents recorded at E = +0.90 V/SCE *vs.* PCP concentration.

Figure 7(a) shows SWV responses recorded with the MWCNT-EP composite electrode for various concentrations of PCP under optimized conditions, *i.e.*, frequency (f) of 10 Hz, modulation amplitude (MA) of 0.1 V, and step potential (SP) of 0.01 V. Figure 7(b) corresponds to the calibration curve of the useful current with PCP concentration, which shows the linear dependence for the concentration interval ranged between 2 and 12 μM PCP. Under the conditions of this applied technique application, the best sensitivity was achieved, which is comparable, however, with the sensitivity obtained by DPV. This aspect should be attributed to the diminution of the capacitive component and implicit the background current that affects the useful signal of the PCP detection by SWV.

Even if the adsorption property of the carbon-based electrode towards the target analyte is not generally desired, because of electrode fouling, this could be exploited however in a positive way, namely to detect PCP at trace levels by applying the preconcetration/voltammetric detection scheme.

**Figure 8.**
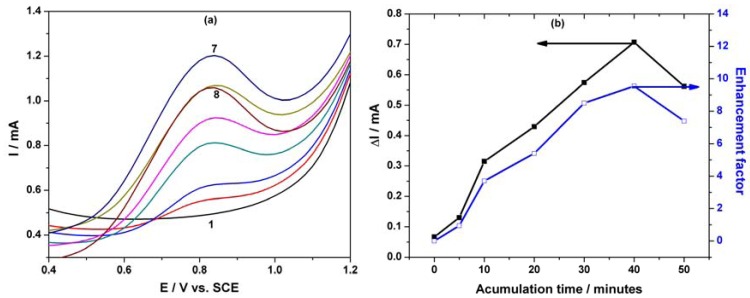
(**a**) DPVs of the MWCNT-EP composite electrode (modulation amplitude of 0.2 V, step potential of 0.02 V), and a potential scan rate of 0.05 Vs^−1^ in the potential range between +0.5 V and +1.2 V *vs.* SCE in 0.1 M Na_2_SO_4_ supporting electrolyte (curve 1) and in the presence of 8 μM PCP concentration after different accumulation times: (2) 0 min; (3) 5 min; (4) 10 min; (5) 20 min; (6) 30 min; (7) 40 min; (8) 50 min. (**b**) Peak current responses and enhancement factors for the detection of 8µM PCP at MWCNT-EP composite electrode as a function of the accumulation time recorded at E = +0.83 V/SCE.

It is well-known that in a preconcentration/voltammetric detection scheme, the extent of preconcentration is a function of accumulation time, which determines the degree of adsorption on the electrode surface. The effect of accumulation time on the currents of the differential-pulsed anodic peaks recorded at +0.83 V/SCE corresponding to PCP oxidation was investigated. The enhancement factor was determined as ratio of the peak current recorded at different accumulation times to that recorded without a preconcentration scheme. The useful oxidation peak currents and the enhancement factors determined for 8 μM PCP using a preconcentration/voltammetric detection scheme at different accumulation times are shown in Figure 8(a).

**Figure 9.**
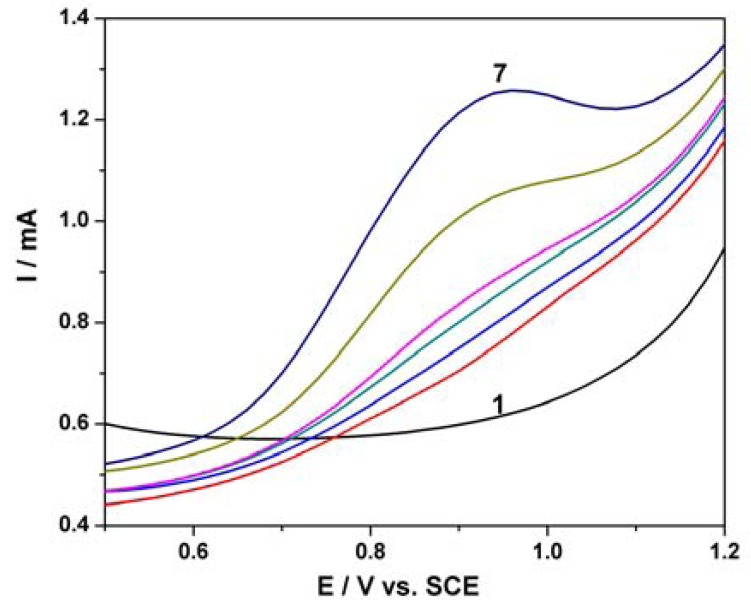
SWVs recorded at MWCNT-EP composite electrode under the optimum conditions of the preconcentration scheme, *i.e.*, accumulation time of 40 minutes with modulation amplitude of 0.1 V, step potential of 0.01 V, and frequency of 10 Hz; potential range between +0.5 V and +1.2 V/SCE in 0.1 M Na_2_SO_4_ supporting electrolyte (1) and in the presence of different PCP concentrations: (2) 0.2 μM; (3) 0.4 μM; (4) 0.6 μM; (5) 0.8 μM; (6) 2 μM; (7) 4 μM.

The oxidation peak currents for this compound increased with accumulation time up to 40 min, which was selected as the optimum accumulation time because a maximum enhancement factor was reached, while at longer accumulation times the peak currents decreased. The enhancement factor value of about 11 at the oxidation potential of +0.83 V/SCE revealed an effective concentration effect of the MWCNT-EP composite electrode on PCP regarding the improvement of its oxidation signal. As a consequence, the accumulation time of 40 minutes was chosen as an optimum time for further square-wave voltammetric experiments. An example of SWV series recorded under the optimum conditions of the preconcentration scheme that assume the accumulation time of 40 min within PCP concentration ranged from 0.2 μM to 4 μM is shown in Figure 9. Applying the above-proposed preconcentration/voltammetric detection using the SWV technique with a modulation amplitude of 0.1 V, step potential of 0.01 V and a frequency of 10 Hz allowed the detection of lower concentrations of PCP with better sensitivity, in comparison with the simple SWV applying without preconcentration. Under these working conditions, a significant enhancement of the electroanalytical parameters of the PCP detection was achieved, *i.e.*, about 10 times better sensitivity and 30 times lower detection limit (see Table 1).

### 3.6. Amperometric Measurements

For practical working applications the optimum analytical procedure should involve the recording of the chronoamperogram, based on the existing well-established essential point of reference provided by the voltammograms. Thus, a series of chronoamperograms was recorded at the potential value of +0.97 V/SCE within a PCP concentration range between 2 and 10 μM (results are not shown here) and the sensitivity obtained was poor compared to CV, probably due to electrode fouling (see Table 1). An alternative to the amperometric detection to improve the electroanalytical parameters and proposed in this work, is the use of MPA with three potential pulses, whose values were established based on CV behaviour. The pulses were applied continuously using the following scheme:

1. +0.97 V/SCE for a duration of 50 ms, where PCP is directly oxidized on the electrode surface,
2. +1.25 V/SCE for a duration of 50 ms, considered as cleaning potential because O2 evolution occurred,
−0.1 V/SCE for a duration of 50 ms, where the reduction process involving the electrode surface occurred.

**Figure 10.**
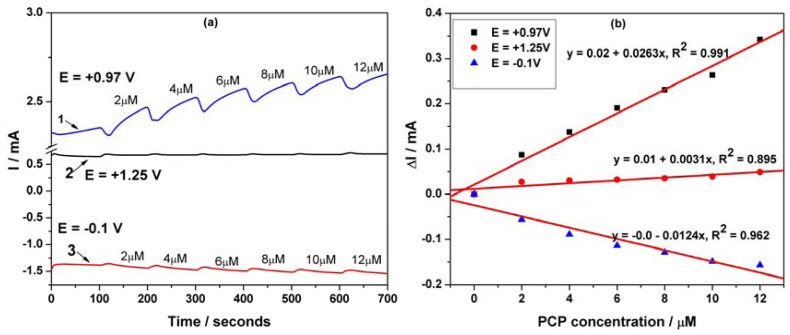
(**a**) Multiple-pulsed amperograms (MPAs) of the MWCNT-EP electrode in 0.1 M Na_2_SO_4_ supporting electrolyte and in the presence of different PCP concentrations: 2, 4, 6, 8, 10 and 12 μM recorded at (1) E = +1.25 V; (2) E = +0.97 V and (3) E = −0.1 V *vs.* SCE.(**b**) The calibration plots of the currents recorded at (1) E = +0.97 V, (2) E = +1.25 V, (3) E = −0.1 V/SCE *vs.* PCP concentration.

Figure 10(a) presents the pulsed amperograms recorded at each potential values for PCP detection. As can be seen, for each potential value the corresponding current depended linearly on PCP concentration (Figure 10(b)), but only the anodic ones have been taken into consideration. Reversing, the advanced oxidation and the reduction processes allow the in-situ regeneration of the electrode surface. Applying the MPA technique improved significantly the performance of the electrode for PCP detection.

These working conditions for applying MPA led to very good sensitivity, quite better than that reached by SWV (without preconcentration step). Also, the best limit of detection (0.006 μM, Table 1) was achieved using this technique, which can be regarded as very suitable for practical application. The electroanalytical parameters for the concentration ranges, where a linear dependence was obtained at the different potential values in relation with the applied electrochemical techniques with or without the preconcentration scheme, are gathered in Table 1. The limit of detection (LOD) was evaluated based on 3 S_B_/b [18], where S_B_ is the standard deviation of the mean value of three voltammograms/amperograms of the blank and b is the slope of the straight line in the analytical curve by using each electrochemical technique. The reproducibility of the electrode using the above-mentioned techniques was evaluated for three replicate measurements of PCP detection as relative standard deviation. A recovery test was performed by analyzing three parallel tap water samples, which contained 2 μM PCP. This test was run in 0.1 M Na_2_SO_4_ as supporting electrolyte and a recovery of 96% with a RSD of 2.8% was found for PCP determination using MPA applied at three potential values, *i.e.*, the first at +0.97 V/SCE for time duration of 50 ms, the second one at +1.25 V/SCE for time duration of 50 ms, and the third one at −0.1 V/SCE for time duration of 50 ms. Finally, the results obtained by this method were compared with those obtained by means of a conventional spectrophotometrical method. Based on the results, it can be concluded that the two methods givd very close results and that the accuracy of the proposed MPA method is good.

## 4. Conclusions

Multi-wall carbon nanotube-epoxy composite electrode was successfully prepared by a two-roll mill procedure, using tetrahydrofuran (THF) solvent that assured a very good dispersion of MWCNT to avoid agglomeration, which was shown by SEM images. The optimum MWCNTs content in the composite material was selected based on the percolation threshold in relation with the electrical conductivity desired for electrochemical application. This composite electrode exhibited electrocatalytic activity towards pentachlorophenol oxidation that is controlled by diffusion, a desired aspect for electroanalysis. The electrochemical determination of PCP at the MWCNT-EP composite electrode was achieved using CV, DPV, SWV, CA, and MPA techniques, which denote different electroanalytical parameters. Under all tested working conditions, this electrode exhibited superiority *vs.* other electrodes, which were reported in the literature [9–11]. Despite the adsorption of PCP on MWCNT is an undesired aspect, this study demonstrated that this property could be exploited in a positive way for PCP determination at the trace level. Under optimized working conditions in relation with the accumulation time and PCP concentration a significant enhancement of the electroanalytical parameters was achieved. However, for the practical application, CA as the simplest electrochemical technique did not allow to obtain good electrochemical detection results, probably because of electrode fouling. MPA applying led to improve the electroanalytical parameters of PCP detection, especially the lowest limit of detection, which makes the composite electrode appropriate for electrochemical determination of a wide range of PCP concentrations in aqueous solution.

## Figures and Tables

**Table 1.** Electroanalytical performance of the MWCNT- EP composite electrode for the detection of PCP in 0.1 M Na_2_SO_4_ supporting electrolyte.

**Peak Potential**	**Technique Used**	**Concentration range (μM)**	**Sensitivity (μA/μM^−1^)**	**Correlation coefficient (*R*^2^)**	**LOD (μM)**	**LQ (μM)**	**RSD** [**] (%)
+0.97 V	CV	2–10	5.3	0.990	1.633	5.443	4.284
+0.80 V	DPV	2–12	11	0.984	0.801	2.671	0.572
+0.90 V	SWV	2–12	16	0.998	0.991	3.306	0.786
+0.95 V	Prec./SWV	0–4	138	0.952	0.033	0.111	0.224
+0.97 V	MPA	2–12	26.3	0.991	0.006	0.021	1.820
+1.25 V			3.1	0.895	0.055	0.185	5.402
+0.97 V	CA	2–10	0.296	0.979	1.547	5.158	2.476

[**] RSD-relative standard deviation was determined for three replicate measurements; In comparison with other electrodes reported in the literature for the PCP detection [9–11], the MWCNT-EP composite electrode exhibited enhanced electroanalytical performance regarding both sensitivity the lowest limit of detection.
